# 

**Published:** 1983

**Authors:** 


					Centenary Year
1883-1983
BRISTOL
MEDICO
CH1RUKGICAL
JANUARY/APRIL 1983
VOLUME 98 (i)
NUMBER 365

				

